# Perceptions of Value and Adoption of ADRD Modifying Drugs Among Physicians Around the U.S.

**DOI:** 10.1093/geroni/igaf122.3992

**Published:** 2025-12-31

**Authors:** Dinithi Perera, Michelle Keller, Zaldy Tan, Chanelle Mizrahi, Nabeel Qureshi, Golnaz Yadollahikhales, Sarah Kremen

**Affiliations:** University of Southern California, Los Angeles, California, United States; University of Southern California, Los Angeles, California, United States; Cedars-Sinai Medical Center, Los Angeles, California, United States; University of Southern California, Los Angeles, California, United States; Cedars-Sinai Medical Centers, Los Angeles, California, United States; Cedars-Sinai Medical Center, Los Angeles, California, United States; Cedars-Sinai Medical Center, Los Angeles, California, United States

## Abstract

Following recent FDA approvals in the U.S. of two anti-amyloid medications for Alzheimer’s Disease, lecanemab and donanemab, we aimed to examine clinicians’ perceptions of the value of and real-world adoption experiences with prescribing these medications. While these monoclonal antibody treatments are the first medications to clear amyloid in the brain, they come with significant treatment burdens and risks. Our objective was to better understand the risk-benefit evaluations physicians make when selecting candidates for treatment; the practice-level, insurance, and health system obstacles they’ve had to navigate when implementing these complex medications into their practices; and how they communicate the risks and benefits to patients and their families. Using purposive and snowball sampling, we interviewed 10 physicians including geriatricians, neurologists and psychiatrists to understand the drivers of and barriers to adoption of these medications by physicians. We identified 5 themes from our qualitative interviews: 1) a broad continuum of wariness to avid proponent of the medications; 2) differences in a propensity to prescribe these medications and approaches to care between different clinical specialties, with strong support from neurologists and a more cautious approach from geriatricians; 3) varied communication styles in how physicians present the risks and benefits with patients and their families, 4) significant variation in practice and health-system implementation, from a planned approach to a less organized approach, and 5) clinicians facing shifting roles of balancing hope with limited data, managing ethical complexity, and bearing the emotional weight of guiding patients through novel therapies.

